# Nursing care missed in patients at risk of or having pressure ulcers

**DOI:** 10.1590/1518-8345.1462.2817

**Published:** 2016-11-21

**Authors:** Jonathan Hermayn Hernández Valles, María Guadalupe Moreno Monsiváis, Ma. Guadalupe Interial Guzmán, Leticia Vázquez Arreola

**Affiliations:** 1 MSc, RN, Hospital Universitario "Dr. José Eleuterio González", Monterrey, NL, México.; 2 PhD, Professor, Facultad de Enfermería, Universidad Autónoma de Nuevo León, Monterrey, NL, México.; 3 MSc, Professor, Facultad de Enfermería, Universidad Autónoma de Nuevo León, Monterrey, NL, México.

**Keywords:** Pressure Ulcer, Nursing Care, Nursing Staff, Attention, Quality Management

## Abstract

**Objective:**

to determine the nursing care missed as perceived by the nursing staff and its relation with the nursing care missed identified in the assessment of patients at risk of or having pressur ulcers.

**Method:**

descriptive correlation study. The participants were 161 nurses and 483 patients from a public hospital. The MISSCARE survey was used in combination with a Nursing Care Assessment Form for Patients at Risk of or having pressure ulcers. For the analysis, descriptive and inferential statistics were used.

**Results:**

the nursing staff indicated greater omission in skin care (38.5%), position change (31.1%) and the registration of risk factors for the development of pressure ulcers (33.5%). The nursing care missed identified in the assessment related to the use of pressure relief on bony prominences and drainage tubes interfering in the patient's movements (both with 58.6%) and the use of pneumatic mattresses (57.6%).

**Conclusion:**

a high percentage of nursing care missed was found according to the staff's perception. Nevertheless, the assessment of the nursing care missed was much higher. No significant relation was found between both. Therefore, it is a priority to reflect on the importance of objective patient assessments.

## Introduction

Around the world, quality and patient safety represent a relevant aspect for hospital systems, due to its ethical and financial impact^1^. The World Health Organization (WHO) estimates that one out of 10 patients living in industrialized countries is a victim of adverse events in health care; this inappropriate patient care causes medical spending on hospitalization, nosocomial infections and inability; in some countries, this costs between 6,000 and 29,000 million dollars per year^2^. Therefore, several national and international entities have participated in the formulation of strategies to maintain high quality standards. In Mexico, as from 1999, the quality model for patient safety has been developed, which consists of basic actions for patient safety, critical systems, patient-centered care and organizational management. The creation and development of this model is intended to improve the quality of the care services and of the safety offered to the patients, mainly while in hospital^3^. Patient safety is defined as avoiding and preventing errors in the care delivered at the health services, with a view to avoiding adverse events that put the users' life at risk. In addition, it is a priority component of the care quality, in which the nursing staff plays an important role, being one of the main care providers^4^.

While in hospital, a number of adverse events can take place as a result of care. These events are damages a procedure causes to the patient, whose results cannot be attributed to the disease or health condition that led to the search for care^4^. These errors, as products of the care provided, for example the administration of a wrong dose to the patient, are called commission errors; nevertheless, in practice, procedures also exist that are not accomplished according to the patient's demands, such as help with walking. These are called omission errors. According to the Agency for Healthcare Research and Quality, they are harder to acknowledge than an execution error, therefore representing a greater problem for the patient^5^. 

The nursing care missed is considered an omission error, defined as any aspect of care the patient needs and which is omitted or significantly delayed^6^. Due to some factors, nursing care regularly is not fully performed. The most frequent cases are due to human, material resources and communication resources. Concerning these factors, in the literature, personnel shortage, lack of time required for care, deficient teamwork, the "that's not my job" syndrome, ineffective delegating, habits of leaving pending work, denial to perform the corresponding work and low staffing^5^ ^,^ ^7^ ^-^ ^12^. 

Some studies developed to determine the nursing care missed have demonstrated that walking three times per day, patient education, oral hygiene, changing the patient's position every two hours, bed bathing, skin care, appropriate surveillance and development or updating of nursing care plans are regularly omitted^5^ ^,^ ^7^ ^-^ ^10^. Care omission in practice entails different negative outcomes for the patient, such as increased mortality rates^13^, infections^14^, prolonged hospital stays^14^, pressure ulcers^15^, patient falls^16^, adverse events^17^, post-surgery complications^18^ ^)^ and patient dissatisfaction^19^. Although all care outcomes are relevant, this study is focused on pressure ulcers. 

Pressure ulcers (PU) represent an important challenge professionals face in their care practice, mainly because they can be prevented. One of the first steps to prevent them is the use of a scale to detect the type of risk, which can be low, medium and high. According to the results of the assessment, a relevant care plan should be elaborated to avoid or reduce the development of ulcers. Overall, immobility is considered the main predisposing factor for the development of a PU. A relation with nutrition is also assumed. It is estimated that 95% of the PU can be avoided through appropriate management of the risk factors predisposing to their development^20^.

According to WHO, the global prevalence of PU ranges between 5 and 12%, with 7% in America and no exact data for Mexico; nevertheless, in a study developed at health institutions from the 32 entities of the federation, the rate reported was 12.92%^21^. The development of PU is closely related with the nursing care provided to the patient. It is beyond doubt that, the higher the quality and continuity of preventive care for patients at risk of developing a PU, the lower the incidence rates will be^20^. It has been demonstrated that bedridden patients present at least one pressure ulcer. Its presence was mostly related to the lack of movement, as the patients were hospitalized at intensive care and medical-surgical services. It was also demonstrated that the ulcers were caused by deficient nutrition, as well as by inappropriate management of humidity due to incontinence^22^.

Until date, few studies have measured the nursing care missed and these have focused on the nursing staff's perception, which is relevant because of the negative impact in the patient outcomes. Although the literature recommends linking the care missed with the care outcomes, little has been discussed. Therefore, in this study, it is considered relevant to identify the nursing care missed according to the nursing staff and its relation with the nursing care identified in the assessment of patients at risk of developing PU. Identifying the care granted and missed related to PU and discovering the factors associated is useful for nurse managers, as it provides precise information for the management of resources and care protocols or specific care plans for the prevent of PU. That is mainly relevant because PU can be prevented. 

## Objectives

### General Objective

Determine the nursing care missed according to the nursing staff and its relation with the care missed identified in the assessment of patients at risk of or having pressure ulcers.

### Specific Objectives

Identify the nursing care missed in hospitalized patients at risk of or having pressure ulcers according to the nursing staff. 

Identify the factors why nursing care is missed in hospitalized patients according to the nursing staff. 

Determine the relation between nursing care missed according to the nursing staff and associated factors. 

Identify the care missed through the assessment of hospitalized patients at risk of or having pressure ulcers.

Determine the relation between nursing care missed according to nursing staff and identified in the assessment of hospitalized patients at risk of or having pressure ulcers.

## Method

A descriptive correlation design was used. The study population consisted of nursing professionals active in direct care delivery to adult patients at medium or high risk of developing PU or having PU and hospitalized at the different services of a public tertiary care hospital in the metropolitan area of Monterrey, Nuevo León, Mexico. To estimate the sample, the statistical software *nQuery Advisor* version 4.0 was used, using a bilateral correlation parameter with 90% power, a mean effect size of .26 and significance .05, resulting in an estimated sample of 161 nurses. Three patients were randomly selected from each nurse (*n*= 483) for the sake of more representative assessments, as patients at medium or high risk of PU or having PU were considered and care should be provided in accordance. To measure the nursing care missed, the Nursing Care Missed (*MISSCARE*) survey was applied to the nursing staff, which consists of 54 statements, divided in three parts^23^. The first part consists of demographic and job data of the nursing professionals, totaling 13 statements; the second of the nursing care elements provided to the patient, with 29 statements. In this study, only 13 statements on nursing care for patients at risk of or having PU were used. The third part includes the factors why nursing care is omitted, totaling 17 statements, divided in three classifications: human, material and communication resources. To profile the participating patients, a Patient Identification Data Form was used. To assess the nursing care in patients at risk of or having PU, a form was specifically designed for this study, including the nursing care needed for hospitalized patients at risk of or having PU, according to guidelines of best practices and care protocols for the prevention and management of PU^22^ ^,^ ^24^. 

To collect the information on the nursing staff, the different services were contacted, requesting the staff's voluntary participation. The tool was applied in a reserved space, making sure at all times not to interfere in the nursing care. Next, each nurse's patient notes were revised and patients at medium or high risk of developing PU or having PU were identified, randomly selecting three patients from each nurse. Each patient was consulted for the sake of voluntary participation. If they accepted, the data form was completed, followed by the assessment of nursing care in patients at risk of or having PU, making sure at all times not to interfere in the nursing care or in the patient's meals and sleep. The study complied with the ethical guidelines of the Mexican Law (*Reglamento de la Ley General de Salud en Materia de Investigación para la Salud*)^25^. Approval was obtained from the Research and Research Ethics Committees at the School of Nursing of *Universidad Autónoma de Nuevo León*. The participants gave their informed consent and their dignity, privacy, wellbeing and rights were respected at all times.

To analyze the results, SPSS (Statistical Package for the Social Sciences) version 20.0 was used. To determine the general characteristics of the study population, frequencies, percentages, central trends and dispersion measures were used. The nursing care was classified as care granted and missed. The factors contributing to the care missed were divided among human, material and communication resources. Both were grouped using scores from 0 to 100 and analyzed using means, medians, standard deviation and 95% confidence intervals. The highest mean and median scores correspond to higher levels of care missed. For inferential statistics, Spearman's correlation test was used. 

## Results 

According to the characteristics of the nursing staff that participated in the study, women were predominant (64.6%). The main age group varied between 26 and 30 years, followed by 20 to 25 years. As regards education, 41.6% holds a Baccalaureate in Nursing, followed by 35.4% of Nursing Technicians. The largest proportion of the staff works at the Internal Medicine service (23.6%), followed by Post-surgery and Adult Intensive Care (18% and 17.4%, respectively). Concerning the length of experience at the institution, at the service and professional experience, the largest group ranged between one and five years. The night shift was predominant (41%), followed by the morning shift and pilot plan (both 19.9%). 

In Table 1, the nursing care elements to prevent PU in hospitalized patients are displayed. The predominant care granted according to the nursing staff were patient bath (75.2%), help with toileting needs within five minutes after the request (73.9%) and patient assessments per shift (73.3%). The largest proportion of care missed was found in skin care/wound care (38.5%), followed by the registration of factors predisposing to the development of PU (33.5%) and changing the patient's position every two hours or as needed and in the patient discharge plan and education (both 31.1%). The mean coefficient for the care missed was 29.95 (SD= 18.31) on an index ranging from 0 to 100.

**Table 1:** Nursing care perceived by nursing staff corresponding to PU prevention in hospitalized patients. Monterrey, NL, México, 2015

Care elements	Care Provided (ƒ % )	Care Missed (ƒ % )
Change patient's position every two hours or as needed	111 (68.9)	50 (31.1)
Full documentation of necessary data	112 (69.6)	49 (30.4)
Register factors predisposing to the appearance of PU ^†^	107 (66.5)	54 (33.5)
Patient bath	121 (75.2)	40 (24.8)
Patient discharge plan and education	111 (68.9)	50 (31.1)
Advice to patient and family on how to prevent PU	113 (70.2)	48 (29.8)
Assessment of risk factors predisposing to PU	112 (69.6)	49 (30.4)
Establishment of care plan and execution according to risk of PU	116 (72.0)	45 (28.0)
Use of resources available and necessary to prevent PU	112 (69.6)	49 (30.4)
Assessment of patients per shift	118 (73.3)	43 (26.7)
Reassessments of patient according to health condition	115 (71.4)	46 (28.6)
Help with toileting needs within five minutes after request	119 (73.9)	42 (26.1)
Skin care/wound care	99 (61.5)	62 (38.5)

Care elements: †PU - Pressure Ulcers

Table 2 shows the factors that explain why the loss of care, according to the nurses, is mainly due to human resources, with an average of 85.61 (SD=10.33), followed by material and communication resources.

**Table 2:** Indices of factors contributing to nursing care missed. Monterrey, NL, Mexico, 2015

Indices	Mean	Median	SD*	95% confidence interval	
				Lower Limit	Upper Limit
Human Resources	85.61	91.66	10.33	84.00	87.21
Material Resources	82.40	88.88	15.64	79.96	84.83
Communication	81.22	83.33	11.61	79.41	83.02

Type of statistics: ^*^SD - Standard Deviation

The nursing care the nursing staff perceived as missed was negative and significantly related with the factors the staff perceived: human resources (r_s_ = -0.293, p< .05), material resources (r_s_ = -0.363, p< .05) and communication resources (r_s_ = -0.311, p< .05).

What the patients' characteristics are concerned, the mean age was 38.32 years (SD=9.88), ranging from 21 to 81 years. On average, the patients spent 6.95 (SD= 2.66) at the hospital, ranging between one and 21 day. Male patients were predominant (62.5%). As regards the specialty, patients hospitalized in internal medicine were predominant with 23%. 51.6% presented medium risk for the development of PU. It should be highlighted that 26.1% of the patients presented PU in the assessment.

Table 3 shows the nursing care granted and missed in patients considered at risk or having PU. The most observed care granted was related to diaper use with a clean and dry diaper (73.1%), application of dressings in case of PU (57.1%) and absence of zones exposed to humidity due to incontinence (56.9%). The predominant nursing care missed was the use of some kind of pressure relief on bony prominences (58.6%) and drainage tubes without interfering in the patient's movements (58.6%), followed by the patient's positioning with good body alignment (58.2%). The mean score for care missed was 52.01 (SD= 5.71), on an index that ranged from 0 to 100.

**Table 3:** Assessment of nursing care for PU prevention in hospitalized patients. Monterrey, NL, Mexico, 2015

Care elements	Care Provided (ƒ %)	Care Missed (ƒ %)
Absence of zones exposed to humidity due to incontinence	275 (56.9)	208 (43.1)
Absence of dry skin	214 (44.3)	269 (55.7)
Absence of skin redness	225 (46.6)	258 (53.4)
Absence of maceration of the skin	215 (44.5)	268 (55.5)
Absence of humidity in areas like armpits, under the breasts or in skinfolds	272 (56.3)	211 (43.7)
Use of preventive measures in zones in contact with therapeutic devices	262 (54.2)	221 (45.8)
Bed linen is kept dry	250 (51.8)	233 (48.2)
Fixed drainage tubes without interfering in patient's movements	200 (41.4)	283 (58.6)
Patient's position with good body alignment	202 (41.8)	281 (58.2)
Use of pneumatic mattresses	205 (42.4)	278 (57.6)
Use of some kind of pressure relief on bony prominences	200 (41.4)	283 (58.6)
Maintain patient's daily hygiene	208 (43.1)	275 (56.9)
Move the patient at least every two hours	220 (45.5)	263 (54.5)
In case of diaper use, diaper is clean and dry (*ni*=234)	171 (73.1)	63 (26.9)
In case of PU*, application of PU dressing (*ni*=126)	72 (57.1)	54 (42.9)

Care elements: ^*^PU - Pressure Ulcers

To determine the relation between the nursing care the staff perceived as missed and the nursing care identified in the assessment of patients at risk of PU, Spearman's correlation test was applied. The results revealed no significant association (p>.05).

## Discussion 

In this study, it was relevant to identify the care missed according to the nursing staff and assessed in patients at risk of or having PU because the literature indices that care omissions affect the patient outcomes. According to the staff, the main care elements missed are skin care/wound care, assessment and registration of risk factors predisposing to the development of PU, patient education and position change every two hours or as needed. This is in accordance with some authors findings, who mention that the omission of this care increases the risk for the development of PU^5^ ^,^ ^10^ ^-^ ^11^. Therefore, it is relevant for nurse managers to develop PU prevention protocols and the nursing competences needed to put the care plan in practice in accordance with the patient's risk. 

After identifying the care missed, the factors were determined that influence the omission of nursing care according to the staff. These are mainly attributed to human resource factors, followed by material resources and communication. All of these demonstrate a significant and negative correlation with the care lost. Human resources were the most relevant factors the nursing staff considered, highlighting insufficient staff. Different sources have evidenced that a complete and competent staff reduces the omission of care and influences the care outcomes^11^ ^-^ ^12^. When the human resources are limited, the nursing staff prioritizes care and only executes priority patient care, often linked to the medication treatment, which contributes to the risk of developing negative patient outcomes and specifically PU. The second factor the nursing staff considered was related to material resources. When the necessary medications and supplies/equipment are not available or not functioning when needed, the risk of developing some negative patient outcome can increase; in addition, when lacking the equipment needed to perform the interventions according to each patient's health condition, the mortality rates can increase^6^ ^,^ ^12^. Concerning communication, the nursing staff indicates that it is mainly influenced by the unbalanced attribution of patients, too many patients entering and leaving and the unsuitable delivery-reception of patients. That is in accordance with different sources of evidence, mentioning that it is important to improve effective communication among health staff members; communication is crucial for the continuity of patient care, mainly in processes that require appropriate patient information transfer to avoid omissions that affect the outcomes^11^ ^-^ ^12^. 

With regard to care for patients at risk of or having PU, the assessments showed that the most missed nursing care was the use of pressure relief on bony prominences, fixation of drainage tubes without interfering in the patient's movements, patient positioning with good body alignment, use of pneumatic mattresses and daily patient hygiene^5^ ^,^ ^10^ ^-^ ^11^. According to the findings, during the assessments, patients with PU were found, which mostly developed while in hospital. That confirms the relation between care omission and patient outcomes. The omission of care to prevent PU largely increases their development^21^ ^-^ ^22^.

Despite the high rate of care missed according to the nursing staff, the nursing care missed identified in the assessment was much higher. This can contribute to the lack of statistical association between both. This difference in the identification of the care missed could be due to the fact that the nursing staff subjectively assessed the care delivered to the patients; nevertheless, the objective assessment demonstrated greater care omission in patients at risk of or having PU. This finding arouses reflections on the importance of objective assessments, mainly in patients at risk of developing some kind of complication, such as PU in this case, which can be associated with longer hospitalization, costs, rehospitalizations etc. The management should take into account the present findings to monitor the nursing care and improve the quality of care. In addition, the PU indicator should be monitored to duly identify the areas of opportunity and standardize the interventions needed in patients according to their risk of developing PU. 

## Conclusion

The study findings revealed that nursing care is omitted according to the patients' needs while in hospital and that this leads to negative outcomes, including the development of PU. It is important to highlight that the care missed according to the nursing staff was lower than in the assessment of patients at risk of or having PU. That is relevant because studies about care missed have focused on the nursing staff's perception, but rarely on the patient's perception. In this study, however, the perception was contrasted with the assessment of the patients and the results manifest that the assessment is an objective measure to identify the care omissions and their effect on the patient outcomes more precisely.

Among the factors associated with the care missed, the nursing staff highlighted human resources, followed by material and communication resources. This is relevant for the nurse managers, who should implement administrative measures to strengthen the human resources inside the organizations, with specific quantities and competences to deliver continuing care according to the patients' needs and avoid the care missed and its impact on the care outcomes.
